# Lightning Rods

**Published:** 1871-10

**Authors:** 


### LIGHTNING RODS

Sometimes do more harm than good, in causing a house to be "struck with lightning" instead of warding it off.

A copper lightning rod is six times more efficacious than iron.

Iron conducts lightning one thousand million times better than water.

The thicker a lightning rod is, the more efficacious it is. The longer a rod is, the more imperfectly does it conduct.

The end of the rod which is in the earth should extend far enough down to end in a layer of earth which is always damp or wet, and that damp layer should be as extensive at least as the area of the building.

